# Granulocyte colony-stimulating factor increases the therapeutic efficacy of bone marrow mononuclear cell transplantation in cerebral ischemia in mice

**DOI:** 10.1186/1471-2202-12-61

**Published:** 2011-06-24

**Authors:** Xue-Mei Zhang, Fang Du, Dan Yang, Rui Wang, Chun-Jiang Yu, Xiang-Nan Huang, Hong-Yan Hu, Wei Liu, Jin Fu

**Affiliations:** 1 Department of Neurology, Second Affiliated Hospital, Harbin Medical University, Harbin, 150086, China; 2 Department of Science Research Management, Second Affiliated Hospital, Harbin Medical University, Harbin, 150086, China; 3 Center for Experimental Research, Second Affiliated Hospital, Harbin Medical University, Harbin, 150086, China

## Abstract

**Background:**

Bone marrow mononuclear cell (BMMC) transplantation is a promising therapy for cerebral ischemia; however, little is known if its therapeutic efficacy may be improved by co-administration of potential modulatory factors in vivo. To explore this possibility, the present study examined the effect of BMMCs and G-CSF on cell proliferation, early neuronal development and neurological function recovery in experimental cerebral ischemia relative to controls that received neither treatment.

**Result:**

Ischemia/infarct area was significantly reduced in BMMCs+G-CSF group relative to animal groups treated with BMMCs only, G-CSF only or saline. Transplanted BMMCs were found to colocalize with the proliferative cell nuclear antigen (PCNA) and the immature neuronal marker doublecortin (DCX). The BMMCs+G-CSF group showed increased numerical density of cells expressing PCNA and DCX, improved performance in adhesive sticker removal test and reduced neurological function severity scores relative to other groups in a time-dependent manner.

**Conclusion:**

BMMCs and G-CSF co-administration exhibits synergistic beneficial effect over time. This effect could be at least partially related to increased proliferation and differentiation of bone marrow stem cells and enhanced host brain regeneration and functional recovery. The results suggest that G-CSF can increase the therapeutic efficacy of BMMCs transplantation in an experimental mouse model of cerebral ischemia.

## Background

Bone marrow mononuclear cells (BMMCs) are a population of pluripotent progenitor cells with potential therapeutic value to boost cell repair and regeneration in some disease conditions. In the context of neurology, BMMCs have been shown to promote neuronal regeneration in ischemic cerebrovascular diseases [1,2]. BMMCs can be modulated by various cellular or hormonal factors, suggesting that their putative therapeutic efficacy might be enhanced via co-administration of these factors in vivo. Granulocyte colony stimulating factor (G-CSF) is one of such biological modulators, and has been shown to enhance proliferation and mobilization of BMMCs in some disease models [3]. For instance, Jin et al [4] have demonstrated that G-CSF promotes the migration of bone marrow cells into injured mouse liver. We have previously shown that transplanted BMMCs may relocate to the cerebrum following ischemic injury [5]. Accordingly, we hypothesize that G-CSF can promote BMMCs homing to ischemic cerebrum, and improve histological outcome and neurological function recovery. To explore these possibilities, BMMCs were isolated from the bone marrow of BALB/c mice, double labeled in vitro with the membrane fluorescent dye PKH26 and the nuclear fluorescence probe 4', 6-diamidino-2-phenylindole (DAPI). These pre-labeled BMMCs were transplanted into host mice received either G-CSF or vehicle following experimental cerebral ischemia. Therapeutic effects were evaluated by assessing infarct size, integration of transplanted BMMCs in the brain, recovery of neurological function.

## Results

### Flow cytometric analysis of donor BMMCs

The BMMC fraction of bone marrow can be a mix of different cellular populations. We characterized BMMCs harvested from the donor mice to obtain their surface antigen profile by fluorescence-activated cell sorting (FACS). The marrow cell markers CD34, CD44, CD45 and Sca-1 were used to label BMMCs, and the percentage of each type were estimated. In average, donor BMMCs exhibited CD34, CD44, CD45 and Sca-1 antigen signals at 93.5%, 45.8%, 80.9% and 1.9%, respectively (Table 1). These data are consistent with the signature surface antigen profile of bone marrow stem cells reported by Okada et al [6].

**Table 1:** Surface antigen profile of donor BMMCs by flow cytometry

Marker	Mouse#1(%)	Mouse#2(%)	Mouse#3(%)	Average (%)	S.E.M
CD34	94.4	90.3	95.7	93.5	2.3
CD44	47.7	45.2	44.6	45.8	1.3
CD45	80.1	79.5	83.2	80.9	1.6
Sca-1	1.4	2.6	1.8	1.9	0.5

### Effect of G-CSF and BMMCs on cerebral infarct size

**Figure 1:**
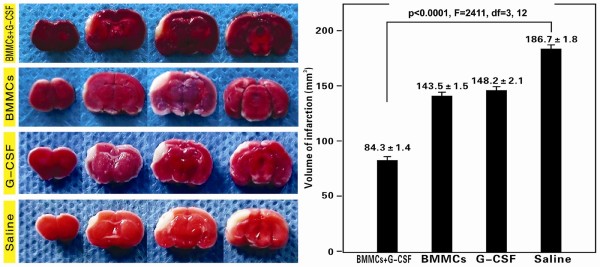
**Representative images of TTC-stained brain slices and infarct volume analysis at 28 days post middle cerebral artery occlusion**. Representative brain slice images (left) were taken at 24 hours following TTC staining. Bar graph on the right shows corrected infarct volumes (CIV) among 4 animal groups. For each brain the CIV value was calculated from slices at 2-mm intervals. Mean infarct volumes are reduced in BMMCs+G-CSF groups (84.3 ± 1.4) relative to BMMCs group (143.5 ± 1.5), G-CSF group (148.2 ± 2.1) and Saline group (186.7 ± 1.8); as well as between the BMMCs or G-CSF group relative to the saline group (Bonferroni's post-tests).

In the present study, effect of BMMC transplantation and G-CSF co-administration following experimental cerebral ischemia was comparatively studied among 4 groups of mice. Two groups of MCA occluded animals received tail vein infusion of BMMCs with (BMMCs+G-CSF group) and without (BMMCs group) G-CSF co-administration. Two other groups of MCA occluded animals were not given BMMC transplantation but received either G-CSF (G-CSF group) or saline (saline group) injection to serve as non-transplant controls (S-Figure 1). We assessed gross cerebral infarct volume among the 4 groups of animals. The mean infarct volume (84.3 ± 1.4 mm^3^) in the BMMCs+G-CSF group was significantly smaller than that in the BMMCs (143.5 ± 1.5 mm^3^), G-CSF (148.2 ± 2.1 mm^3^) and saline (186.7 ± 1.8 mm^3^) groups (p < 0.0001, F = 2411, df = 3, 12; one-way ANOVA analysis) (Figure 1). Bonferroni's multiple comparison tests indicated a statistically significant difference between the BMMCs (p < 0.001) and G-CSF (p < 0.001) groups relative to controls. Also, the infarct size in the BMMCs group was smaller relative to G-CSF group (p < 0.05).

### Effect of G-CSF and BMMCs on PCNA expressing cells

**Figure 2:**
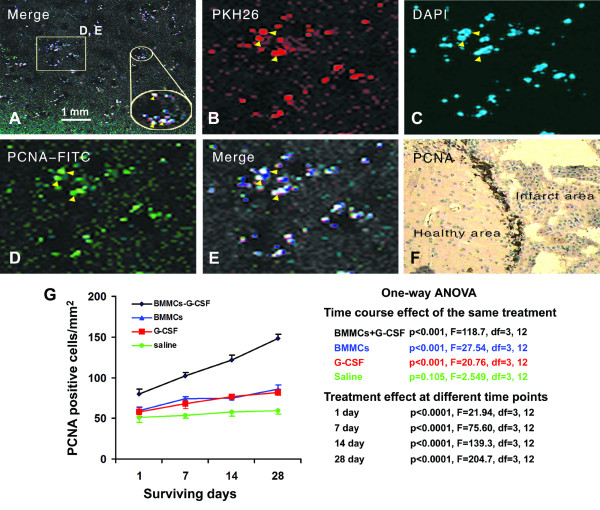
**Colocalization of transplanted BMMCs with PCNA and densitometry of PCNA expressing cells at various surviving times**. Panels A to E are representative confocal images showing that transplanted BMMC, pre-labeled by PKH26 (red) and DAPI (blue), co-localize with proliferative cell nuclear antigen (PCNA, green). (F) illustrates PCNA immmunoreactive cells visualized with the DAB method around the infarct border of the infarct area. (G) shows semi-quantitative data of PCNA positive cells at different time points in the 4 groups of animals. ANOVA table on the right denotes statistical analysis data on the time course effect (longitudinal factor) in the 4 animal groups as well as treatment effect (transversal factor) at the 4 time points.

PKH26-labeled cells emit red fluorescence under 551 nm light excitation, whereas the blue fluorescence of DAPI is detectable using 405 nm light excitation. Thus, donor BMMCs double-labeled in vitro by PKH26 and DAPI can be reliably located in the host brain. Overall, no any PKH26 or DAPI-labeled cells were detected in the brains of mice that did not receive BMMC tail infusion (data not shown, but see Figure 2 and Figure 3 in Zhang et al., 2010)[5]. On the contrary, PKH26 and DAPI double labeled cells were found in mice with BMMC transplantation.

**Figure 3:**
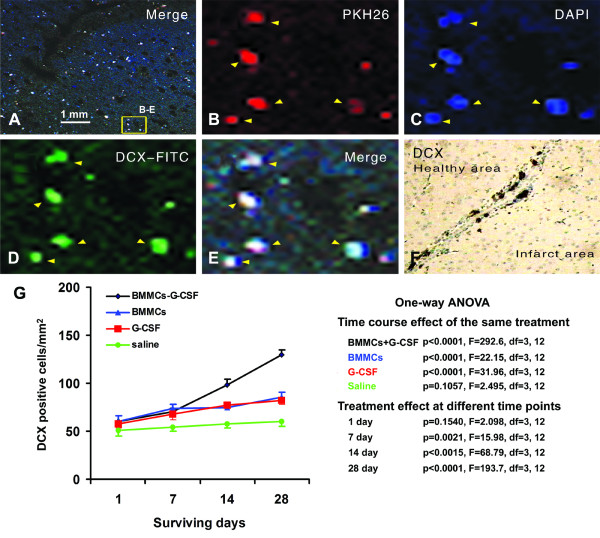
**Colocalization of transplanted BMMCs with DCX and densitometry of DCX expression at various surviving times**. Panels A to E show that transplanted BMMC pre-labeled by PKH26 (red) and DAPI (blue) co-localize with doublecortin (DCX, green) in the cerebral cortex of a recipient mouse (14 days surviving). (F) demonstrates DCX immunoreactivity visualized with the DAB method localizing around the border of the infarct area. (G) shows semi-quantitative measurements of DCX positive cells in MCA occluded mice at different time points. ANOVA table on the right summarizes statistical analysis data on the time course effect (longitudinal factor) in the 4 animal groups as well as treatment effect (transversal factor) at the 4 time points.

One potential effect of G-CSF on BMMCs may involve the proliferative capability of these transplanted cells. We therefore assessed PKH26/DAPI cells co-expressing PCNA, a nuclear cell proliferation marker [7], by confocal fluorescent microscopy. PKH26, DAPI and PCNA triple-labeled cells were found largely around the border of the ischemic and healthy cortical areas (Figure 2A-E). In order to compare cell proliferative rate among the 4 animal groups, we semi-quantitatively assessed the abundance of PCNA labeled cells at day 1, 7, 14 and 28 after MCAO by immunohisochemistry using DAB as a chromogen (Figure 2F). The density of PCNA positive cells was significantly higher in the BMMCs+G-CSF group relative to other groups at the 4 surviving time points (Figure 2G). Bonferroni's multiple comparison tests indicated that at the 1 day surviving point a statistically significant difference existed between BMMCs+G-CSF group and the remaining 3 groups, but not between the BMMCs, G-CSF and saline groups. At other surviving points, there was statistically significant difference between the saline group and each of the treatment groups (the BMMCs+G-CSF, BMMCs or G-CSF group). In addition, the density of PCNA labeled cells was increased with time in the BMMCs+G-CSF (p < 0.001, F = 118.7), BMMCs (p < 0.001, F = 27.54) and G-CSF (p <0.001, F = 20.76) groups, but this change was not found in the saline group (p = 0.105, F = 2.55) (Figure 2G).

### Effect of G-CSF and BMMCs on DCX expressing cells

Recent studies suggest that transplanted BMMCs may transdifferentiate into neurons in ischemic or traumatized brain [8,9]. We have previously shown that PKH26 pre-labeled BMMCs may colocalize with doublecortin (DCX), a marker of immature neurons, in the ischemic host cerebrum [5]. In the present study, we confirmed that PKH26/DAPI double-labeled BMMCs could be co-labeled for DCX in animals surviving 14 and 28 days (Figure 3A-E). In both immuofluorescent and DAB (Figure 3F) preparations, DCX positive cells occurred mostly around the infarct border.

We carried out densitometric analysis of DCX labeled cells (in DAB preparation) in the 4 groups of animals at day 1, 7, 14 and 28 post MCAO (Figure 3G). The density of DCX-labeled cells increased with surviving time in the BMMCs+G-CSF, BMMCs and G-CSF groups (p < 0.0001, one-way ANOVA analysis), but not in the saline group (p = 0.1057). At the 7, 14 and 28 days surviving time points, the density of DCX+ cells in the BMMCs+G-CSF, BMMCs and G-CSF groups was higher relative to the saline group (p = 0.021 to p < 0.0001). Bonferroni's multiple comparison tests also showed statistically significant difference between the saline group and each of the other groups, and between the BMMCs+G-CSF group and the BMMCs or G-CSF group at 14 and 28 surviving days (Figure 3G).

### Effect of G-CSF treatment on neurological function recovery

**Figure 4:**
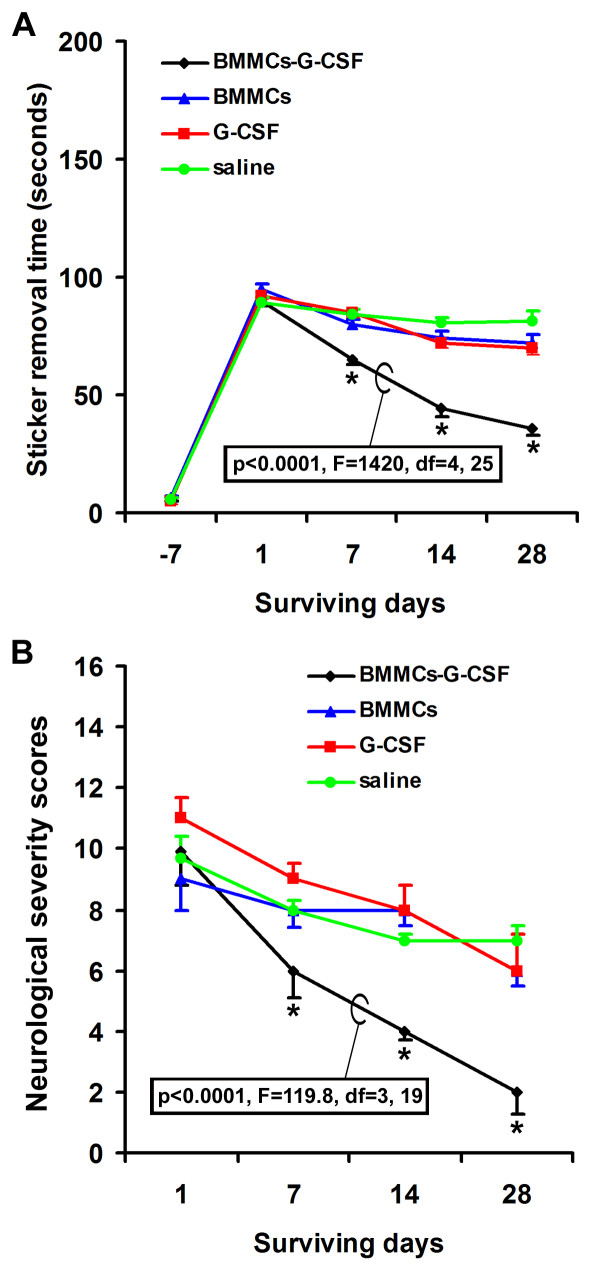
**Neurological evaluation of mice prior to and after middle cerebral artery occlusion**. The upper graph (A) shows the average time scores for adhesive-removal in the 4 animal groups at various surviving points. Mice with BMMCs transplantation combined with G-CSF administration spend less time to remove the adhesive paper on their forearm relative to other groups. The low graph (B) plots the modified neurological severity scores (mNSS) in the 4 animal groups at different time points. The extent of neurological function impairment is significantly less in the BMMCs-G-CSF group relative to other groups, and also reduces with increasing surviving time overall. No differences exist before and at day one after ischemic lesion for either adhesive removal or mNSS scores.

Overall, the time a mouse spent to remove the adhesive paper was dramatically increased after relative to before MCAO. Thus, at 1 day after MCAO, the adhesive removal times was longer but comparable among the 4 groups of occluded animals relative to pre-surgical basal scores (Figure 4A). During the recovery phase, BMMC transplanted mice received G-CSF used less time to remove the adhesive papers as compared to other groups at 7 to 28 days after MACO (Figure 4A) (p < 0.05 to p < 0.001, as determined by Bonferroni's multiple comparison tests in one-way ANOVA analysis). Also, the BMMCs group spent increasingly less time to remove the sticker during the recovery phase (p < 0.0001, F = 1420, df = 4, 25, one-way ANOVA).

The neurological severity scores (NSS) were high but similar (p > 0.05) among the 4 animal groups as assessed at day 1 after MCAO (Figure 4B). During the recovery phase, the scores showed a trend of reduction with time in all groups. The BMMCs+G-CSF group exhibited significantly lower scores relative to the BMMCs only group as well as the two animal groups that did not receive the bone marrow cells at 7 to 28 days surviving points (p < 0.05 to p < 0.001, Bonferroni's multiple comparison tests). The time-dependent decline in neurological severity scores was confirmed by statistical analysis (p < 0.0001, F = 119.8, df = 3, 19, one-way ANOVA).

## Discussion

### Transplanted BMMCs proliferate and transdifferentiate in ischemic cerebrum

Bone marrow stem cell therapy has merged as a promising treatment for acute brain trauma. In practice, donor bone marrow cells are a mix of cellular components, with the BMMC fraction being considered a key player in the beneficial therapeutic efficacy in neuronal recovery. Our fluorescence activated cell sorting analysis suggests a phenotype of the transplanted cells being hematopoietic stem cells in nature, based on their expression of CD34 (93. 5%), CD44 (45.8%), CD45 (80.9%) and Sca-1(1.9%) [10]. The use of PKH26 and DAPI double fluorescent labeling allow us to trace the homing of BMMCs in the recipient mouse brain. Consistent with an early report [11], immunofluorescent co-labelings for PCNA and DCX suggest that some of these pre-labeled BMMCs are undergoing proliferation and appear to trans-differentiate into immature neurons in the ischemic cerebrum.

### BMMCs and G-CSF exhibit synergistic effect on cell proliferation and early neuronal development in ischemic cerebrum

Improving the therapeutic efficacy of BMMC transplant may be of future clinical relevance. Data from the present study support that G-CSF, a biological regulator of BMMCs in vivo, may exhibit beneficial modulatory effect on both the transplanted bone marrow cells as well as the histological and neurological outcome in the host animals in an acute model of cerebral ischemia or infarction. Densitometric analyses show that the amount of PCNA and DCX-expressing cells are increased in the cerebral cortex in G-CSF-treated recipient mice relative to the BMMCs and G-CSF groups, in addition to the saline group. Therefore, it appears that BMMCs transplantation and G-CSF administration each could promote cell proliferation and DCX expression in the ischemic cortex [12], while BMMCs and G-CSF co-treatment elicits a synergistic effect. These findings suggest that BMMCs and G-CSF are both positive modulatory factor in the homing and potential neuronal trans-differentiation of hemopoietic stem cells in ischemic cerebral cortex.

### BMMCs and G-CSF exhibit synergistic effect on histological and neurological outcomes

G-CSF administration appears to also reduce the cerebral infarct size according to our assessment on brain slices. In fact, this effect is most noticeable in animals received BMMC transplant as compared to ischemic animals without BMMC treatment. Thus, BMMC infusion and G-CSF treatment appear to exhibit synergeric protective effect in lesioned host animals. In parallel with the improved histological outcome, animals received G-CSF treatment show better neurological function recovery.

### Mechanistic consideration for the effect of BMMCs and G-CSF in ischemic cerebrum

The precise mechanism by which G-CSF may influence transplanted and host BMMCs, or other cellular components, in the ischemic mouse brain remains to be elucidated. However, a large body of basic and clinical studies suggest that G-CSF can mobilize hemopoietic stem cells, therefore might improve their delivery and differentiation in bodily tissue [12-14]. It is known that G-CSF can promote cell proliferation including stem cell proliferation and their differentiation into neutrophils, eosinophils and mononuclear macrophages. This pro-proliferative or cell trophic effect is probably mediated by G-CSF receptor (G-CSF-R), which is especially enriched on the cell surface of bone marrow stem cells [15,16]. G-CSF-R activation initiates signal transduction through proteins including Jak, Lyn, STAT and Erk1/2, which may cascade cell proliferation, differentiation and maturation [17,18]. Via such a mode G-CSF may help transplanted or host blood stem cells home to the injured brain and potentiate neuronal regeneration [4,19]. Of note, some authors suggest that G-CSF may facilitate the migration and localization of neuronal stem cells into injured areas [20]. Therefore, G-CSF administration might also play a role in promoting intrinsic neuronal self-repair in the brain.

In summary, this work demonstrates that G-CSF administration facilitates the therapeutic efficacy of BMMC transplantation in histological and neurological outcomes in a mouse model of focal cerebral ischemia. Because autologous BMMC transplantation may be useful in the treatment of cerebral injury including ischemia, co-administration of G-CSF may be considered a promising adjunct therapy in clinical practice. Further basic and clinical investigations are warranted to explore the molecular mechanism underlying the effect of combined G-CSF administration and BMMCs transplantation in cerebral ischemia.

## Conclusions

The present study shows that G-CSF may promote the homing, proliferation and potentially neuronal differentiation of transplanted BMMCs in ischemic mouse cerebral cortex. G-CSF administration also appears to improve the efficacy of BMMC transplantation by reducing cerebral infarct size and enhancing neurology function recovery. The data raise a possibility of using BMMC transplantation together with G-CSF administration in clinical treatment of cerebral ischemia.

## Methods

### Experimental animals and reagents

Inbred BALB/c mice at 8 to 10 weeks of age, of male or female gender, with a bodyweight ranging between 18 to 20 g, were purchased from the Animal Center of the Second Affiliated Hospital of Harbin Medical University. The mice were freely accessible to food and water before and after the experimental procedures. Primary antibodies and secondary detection systems, as well as major reagents, used in the present study are listed in Table 2.

**Table 2:** Primary antibodies and major biochemical reagents used in this study

Antibody or Biochemical Reagents	Source	Product #	Dilution
Mouse anti-PCNA antibody	Santa Cruz	BM 0104	1:200
Mouse anti-DCX antibody	Santa Cruz	Sc-2024	1:100
PKH26	Sigma	PKH26PCL	1:1000000
DAPI	Roche	46190	5 μg/ml
Alexa Fluor^® ^488	Invitrogen	A11055	1:200
FITC rat IgG 2α κIsotype	Biolegend	400505	1:200
APC rat IgG 2α κIsotype ctrl	Biolegend	400511	1:200
PE rat IgG 2b κIsotype ctrl	Biolegend	118419	1:200
Percp rat IgG 2b κIsotype ctrl.	Biolegend	B118419	1:200
FITC anti-mouse LY-6A/EC Sca-1	Biolegend	122505	1:100
APC anti-mouse CD34	Biolegend	119309	1:100
PE anti-mouse/human CD44	Biolegend	103007	1:100
Percp anti-mouse CD45	Biolegend	103129	1:100

### Experimental groups

To evaluate the effect of G-CSF administration and BMMC transplantation, mice were divided into donor group (n = 14, for BMMC donation) and recipient group with focal cerebral ischemia. The recipient group was then sub-divided. Thus, Group #1 animals (BMMCs+G-CSF) received BMMCs transplantation combined with G-CSF injection (30 μg/kg/d, i.p.). Group #2 animals (BMMCs) received BMMCs without G-CSF injection. Group #3 animals (G-CSF) received G-CSF injection (30 μg/kg/d, i.p.) without BMMCs treatment. Group #4 was treated with 0.3 ml of saline (0.9% NaCl, i.p.) as vehicle control. For group #1, daily G-CSF injection (30 μg/kg/d, i.p.) started after BMMC transplantation and continued once a day for four successive weeks. The same G-CSF administration protocol was used for group #3 (S-Figure 1). Four animals per group were used for evaluation of infarct size and 4 animals per group were used for histology and immunohistochemistry.

### Isolation, *in vitro fluorescent labeling *and transplantation of BMMCs

As shown previously, BMMCs can be isolated and labeled in vitro prior to transplantation [11,21]. Briefly, the femoral bones were aseptically harvested from donor mice under anaesthesia (sodium pentobarbital, 100 mg/kg, i.p.). After resection, the bone marrow in the medullary cavity was bathed with saline containing heparin (50 U/ml). The bone marrow cells were harvested and suspended in lymphocyte isolation medium under sterile conditions. After dilution with 2 ml phosphate-buffered saline (PBS, 0.01 mol/L, pH = 7.4) at 1:1 ratio, cells were slowly added to yield a relative density of 1.077 g/cm^3^, which was followed by centrifugation at 2000 rpm for 20 minutes. The cells were harvested, washed with PBS three times, and centrifuged at 1200 rpm for 10 minutes. The second layer of cells in the centrifugation tube was removed and re-suspended with DMEM/F12 medium (DMEM/F12, 15% PBS, 100,000 U/L penicillin, pH = 7.4) to provide a cell density > 5 × 10^8 ^cells/L. Then BMMCs were pre-labeled by PKH26 and DAPI according to the manufacturer's instruction [22]. The final density of double-labeled cell suspension was adjusted to 3 × 10^7 ^cells/ml. The viability of BMMCs was greater than 95% measured by trypan blue exclusion. For a given recipient mouse 1 × 10^7 ^double-labeled BMMCs were injected via the tail vein.

### Identification of double-labeled cells by flow cytometric analysis

Putative BMMCs double labeled by PKH26 and DAPI (1 × 10^6 ^cells/ml) were incubated in 2% fetal bovine serum in PBS at 4°C for 20 minutes with 1 μl of mouse monoclonal antibodies specific for CD34, CD44, CD45, Sca-1 or treated with normal mouse serum as a control. Samples were then analyzed by FACS Calibur with CellQuest software (Becton Dickinson, USA).

### BALB/c mice model of focal cerebral ischemia

In order to establish the experimental murine model of focal cerebral ischemia, the middle cerebral artery was occluded with electric coagulation following a modified craniectomy [23]. The surgical procedures have been described previously in detail [11,21].

### Neurological tests

For adhesive removal tests, square-shaped pieces (100 mm^2^) of adhesive-backed paper were used as bilateral tactile stimuli by placing on the distal-radial area of the wrist of each forelimb. Mice were pre-trained for 1 week before tests and assessed basal level of time scores for adhesive removal before MCA occlusion. Mice were tested in 3 trials to measure the mean time to remove the adhesive papers, with a cutoff time of 300 seconds.

The neurological severity scores (NSS) were modified and calculated following the method of Lu D et al [11]. The modified neurological severity scores (mNSS) are given according to animal's performance on motor (muscle status, abnormal movement), sensory (visual, tactile, and proprioceptive) and reflex tests [22,23]. Points reflect the extent of inability to perform a given test; thus, higher scores represent poorer neurological function or presumably greater injury.

### Tissue preparation

Mice were sacrificed and perfused transcardially for 2 minutes at 120 mmHg with 4% paraformaldehyde in 0.01 M phosphate-buffered saline (pH 7.4, PBS) via a left ventricular stab under overdose pentobarbital anesthesia (sodium pentobarbital 100 mg/kg, i.p.). The brains were dissected out and cryoprotected in 30% sucrose at 4°C overnight, embedded in optimal cutting temperature compound, frozen, and prepared into 8-μm-thick frozen sections using a cryostat.

### Histological and morphological evaluation of BMMC homing

To assess the rate of proliferation and survival of cells after BMMC transplantation, a one in eight series of sections from each brain was collected randomly to identify PKH26 and DAPI double positive cells. Additional sections were processed for immunolabeling of PCNA and DCX using microwave antigen retrieval as previously described [11,21]. Briefly, sections (thaw-mounted on slides) were treated with 1% H_2_O_2 _in PBS for 30 minutes, and pre-incubated in 5% normal rabbit serum in PBS with 0.3% Triton X-100 for 30 minutes. Sections were then incubated with PCNA (1:200) or DCX (1:100) antibody. Sections were further reacted with biotinylated rabbit anti-mouse (PCNA) or goat (DCX) antibody at 1:400 concentration (BM0104 Boster company, Santa Cruz Biotechnology ) for 2 hours, and subsequently with the ABC reagent (1:400) (Vector Laboratories, Burlingame, CA) for another 1 hour. The immunoreactivity was then visualized by using DAB (0.05%) and H_2_O_2 _(0.003%). Slides were then dipped in hematoxylin, dehydrated and coverslippered.

For immunofluorescence, sections were incubated with PCNA or DCX antibody overnight, washed extensively and followed by a 2 hours reaction with Alexa Fluor^® ^488 conjugated donkey anti-mouse or anti-goat IgGs (1:200, A21206 Invitrogen, Carlsbad, CA). Sections were then washed and mounted with anti-fading medium before examination on a laser confocal scanning microscope (LSM 510 META; Zeiss, Germany). Sections from every mouse in the recipient group were processed in parallel under identical conditions to minimize variations in staining intensity.

### Data and statistical analyses

All data are presented as mean ± standard deviation. One set of sections from each animal was selected randomly. In each frozen section, 10 microscopic fields were captured randomly at a magnification of 200X. All analyses were processed using the statistical software GraphPad Prism 4. Means were compared using one-way ANOVA with Bonferroni's multiple comparison tests. Statistical significance was set at a p value < 0.05.

## Competing interests

The authors declare that they have no competing interests.

## Authors' contributions

XMZ designed the experiments, established the animal model, performed the histological and morphological evaluations and drafted the manuscript. FD carried out bone marrow mononuclear cell transfusion. XNH and WL performed tissue processing and data analysis. DY and RW participated in the fluorescence-activated cell sorting analysis. CJY and HYH performed the statistical analysis. JF supervised and coordinated the study, and finalized the manuscript. All contributors read and approved the final manuscript.

**Figure 5:**
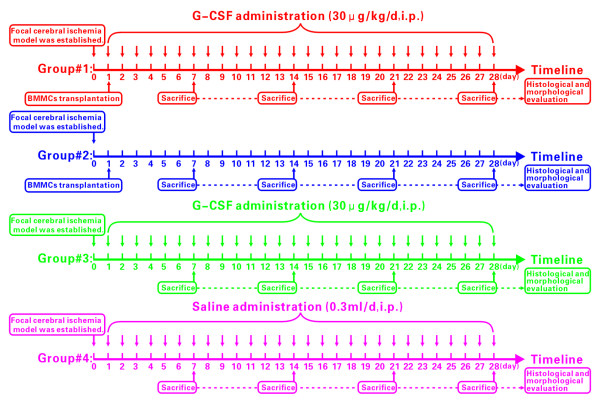
**Experimental design in the study**. Mice with middle cerebral artery occlusion are divided into 4 groups: group#1: mice received G-CSF (30 μg/kg/d, dissolved in 0.3 ml saline, i.p.) for 28 days and BMMC transplantation (1 × 10^7 ^cells dissolved in 0.3 ml saline) via tail vein, one time on the first day after middle cerebral artery occlusion; group#2: mice received BMMC transplantation only (1 × 10^7 ^cells dissolved in 0.3 ml saline); group#3: mice received G-CSF administration alone (30 μg/kg/d, dissolved in 0.3 ml saline, i.p.) for 28 days; and group#4 received 0.3 ml saline (i.p.) for 28 days.
